# Remifentanil patient-controlled versus epidural analgesia on intrapartum maternal fever: a systematic review and meta-analysis

**DOI:** 10.1186/s12884-020-2800-y

**Published:** 2020-03-12

**Authors:** Guolin Lu, Wenshui Yao, Xiaofen Chen, Sujing Zhang, Min Zhou

**Affiliations:** Department of Anesthesiology, Fujian Maternity and Child Health Hospital, Affiliated Hospital of Fujian Medical University, 18 Daoshan Road, Fuzhou, 350001 Fujian Province China

**Keywords:** Remifentanil, Epidural, Fever, Labor, Pain

## Abstract

**Background:**

Intravenous remifentanil patient-controlled analgesia (RPCA) is an alternative for epidural analgesia (EA) in labor pain relief. However, it remains unknown whether RPCA is superior to EA in decreasing the risk of intrapartum maternal fever during labor.

**Methods:**

According to the Preferred Reporting Items for Systematic Reviews and Meta-Analyses (PRISMA) guidelines, a systematic review and meta-analysis was performed by searching PubMed, EMBASE and the Cochrane Central Register of Controlled Trials from inception to April 2019. All randomized controlled trials (RCTs) investigating the risk of intrapartum maternal fever with RPCA compared with EA alone or EA in combination with spinal analgesia during labor were included.

**Results:**

A total of 825 studies were screened, and 6 RCTs including 3341 patients were identified. Compared with EA, RPCA was associated with a significantly lower incidence of intrapartum maternal fever (risk ratio [RR] 0.48, *P* = 0.02, *I*^*2*^ = 49%) during labor analgesia. After excluding 2 trials via the heterogeneity analysis, there was no difference in the incidence of intrapartum fever between patients receiving RPCA and those receiving EA. Satisfaction with pain relief during labor was lower in the RPCA group than that in the EA group (− 10.6 [13.87, − 7.44], *P* < 0.00001, *I*^*2*^ = 0%). The incidence of respiratory depression was significantly greater in the RPCA group than that in the EA group (risk ratio 2.86 [1.65, 4.96], *P* = 0.0002, *I*^*2*^ = 58%). The incidence of Apgar scores < 7 at 5 min in the RPCA group was equivalent to that in the EA group.

**Conclusion:**

There is no solid evidence to illustrate that the incidence of intrapartum maternal fever is lower in patients receiving intravenous RPCA than in patients receiving EA.

## Background

Intrapartum fever in women receiving epidural analgesia (EA) is associated with a higher risk of cesarean delivery and assisted ventilation in neonates, neonatal hypotonia, unnecessary neonatal antibiotic treatment, and even early-onset seizures [1–4]. Intrapartum fever is referred to as maternal temperature not less than 38 °C (100.4 °F) during labor [5]. Epidural-related maternal fever (ERMF) has been reported in approximately 20% of women receiving EA for labor pain relief [5]. A prospective cohort study published that a rise in maternal temperature is associated with a higher body mass index value and longer time from rupture of membranes to delivery and that EA had no this effect on maternal temperature [6].

However, accumulating evidence has confirmed that EA is independently responsible for maternal temperature elevation during labor [1, 5, 7–9]. During epidural labor analgesia, a regular intermittent bolus produced an incidence of ERMF equivalent to that of continuous infusion [10]. Antibiotic prophylaxis has been demonstrated to be ineffective for ERMF [9]. It has been reported that higher levels of the maternal pyrogenic cytokine, interleukin (IL)-6 were measured in intrapartum patients receiving EA than in other patients [5]. Bupivacaine has been found to impair the ability of anti-pyrogenic IL-1r∂ to promote ERMF during labor [7]. Multiple inflammatory signaling pathways are activated by ropivacaine in human umbilical vein endothelial cells and placental trophoblasts as a result of ERMF [11]. Obviously, ERMF is likely to be associated with noninfectious inflammation.

Remifentanil has been found to ameliorate the inflammatory response induced by surgical stress in rats [12]. It is likely that the anti-inflammatory effect of remifentanil may have bring a potential benefit for intrapartum maternal fever. Remifentanil is commonly used in the obstetric analgesia. Recently, a multicenter, randomized controlled trial (RCT) concluded that intravenous remifentanil patient-controlled analgesia (RPCA) is more efficient than intramuscular pethidine for pain relief during labor [13]. Although RPCA is inferior to EA regarding its efficacy in attenuating labor pain [14, 15], Douma et al., published that pyrexia developed to a lower degree in parturient women receiving RPCA compared with patients receiving EA [16]. A similar tendency has also been observed in a randomized multicenter equivalence trial [14]. Inconsistent with these findings, another trial has provided the evidence that there is no difference in the intrapartum fever between patients receiving RPCA and those receiving EA [17]. It still remains controversial whether RPCA is superior to EA in decreasing the rate of the intrapartum maternal hyperthermia.

Intravenous RPCA is shown to be an alternative to pain relief during labor [18]. Currently, there is no review aimed at assessing the risk of an intrapartum maternal fever between patients receiving RPCA and those receiving EA. We hypothesized that the incidence of intrapartum fever in patients receiving RPCA is lower than that in those receiving EA. To test this hypothesis, we performed a meta-analysis of RCTs evaluating the risk of intrapartum fever and the efficacy and safety of intravenous RPCA in women compared with EA in women.

## Methods

### Search strategy

A systematic review was conducted in line with the Preferred Reporting Items for Systematic Reviews and Meta-Analyses (PRISMA) [19] guidelines before the study was designed. The study protocol was registered prospectively in the International Prospective Register Of Systematic Reviews (PROSPERO). (https://www.crd.york.ac.uk/PROSPERO/#myprospero ID = CRD42019135235). A systematic literature search was carried out in the PubMed, EMBASE, and the Cochrane Central Register of Controlled Trials for publications published before April 30, 2019. No language limitations were applied. All were restricted to title and abstract. We also queried coauthors about nonpublished studies and manually scanned reference lists of relevant reviews, trials, and reports in case of unknown sample size. Otherwise, we did not contact authors for further information since we strived to decrease reporting bias. The search terms were “analgesia,” “analgesic,” “pain,” “intrapartum,” “maternal,” “maternity,” “labor,” “parturition,” “delivery,” “parturient,” “fever,” “heat,” “hyperthermia,” “temperature,” “pyrexia,” and “remifentanil” (examples of the online search strategy are shown in the supplementary text).

### Identification of eligible studies

Inclusion criteria included English articles regarding RCTs, studies comparing intravenous RPCA with EA during labor, an epidural infusion of at least 4 h, and intrapartum fever defined as a maternal temperature ≥ 38 °C. All women who were in labor with an American Society of Anesthesiology class I or II status, a planned vaginal delivery, and a gestation of at least 32 weeks of gestation, and who were aged not less than 18, were considered to be eligible for inclusion. Studies were eligible for inclusion if they reported one or more of the following predetermined outcomes: our primary outcome was intrapartum maternal fever (temperature ≥ 38 °C) after labor analgesia; secondary outcomes included the satisfaction with regard to pain relief during labor expressed as the area under the curve (AUC), respiratory depression, and the Apgar score at 5 min.

Studies in line with any of the following criteria were excluded: nonhuman studies, articles in other than English, review articles, conference abstracts, letters, editorial articles, irrelevant topics, nonrandomized trials, incorrect comparisons, or the primary outcomes not reported. Studies were excluded if they enrolled pregnant women who had a body mass index ≥40 kg/m^2^, severe preeclampsia, or an initial maternal temperature ≥ 38 °C, or who used antibiotics during labor.

### Assessment of methodological quality

Two authors, Wenshui Yao, and Xiaofen Chen, were responsible for assessing the methodological quality of the included studies, according to the Cochrane Collaboration tool. Studies were screened on random sequence generation and allocation concealment, blinding of participants and personnel, blinding of outcome assessment, incomplete outcome data, selective reporting, and other bias based on the sample size calculation. The studies were ranked with a low, high, or unclear risk of bias.

### Data extraction

Two reviewers (Lingshan Ye and Wenshui Yao) used a predefined form to independently extract data regarding study characteristics (time period, country, maternal age, gestational age, and labor analgesia regimen), participant number (randomized, dropped-out, crossed-over, actually received pain relief), and the definition and measured duration of intrapartum maternal fever.

### Data synthesis and analysis

All analyses were carried out using Review Manager (RevMan version 5.3.5, Copenhagen: The Nordic Cochrane Center, The Cochrane Collaboration, 2014). We calculated risk ratios and 95% confidence intervals using the random effects Mantel-Haenszel model for dichotomous outcomes. For continuous outcomes, we calculated weighted mean differences and 95% confidence intervals. The significance level was set as *P* < 0.05. I^2^ was used to assess heterogeneity. When the I^2^ value was larger than 50% (≥ 50%), a sensitivity analysis was conducted to explore the influence of measure duration and group allocation. Publication bias was examined using a funnel plot.

## Results

### Study identification and characteristics

**Fig. 1:**
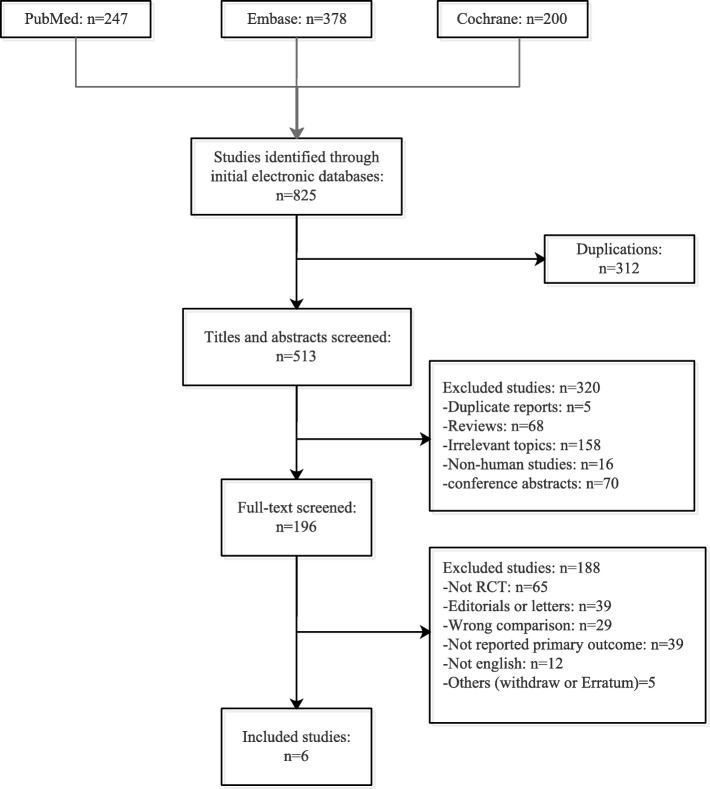
Study selection. RCT = randomized controlled trial

The flow of the study selection is shown in Fig. 1. The systematic search yielded a total of 825 studies. After removing duplicates, the title and abstract of 513 studies were screened. Subsequently, 196 full articles were evaluated for eligibility. At the end of the selection, six RCTs enrolling 3341 participants undergoing delivery were included in the meta-analysis [14–17, 20, 21] (major characteristics reported in Table 1). We excluded 1 multicenter trial only comparing intravenous remifentanil with intramuscular pethidine, 39 trials not reporting intrapartum maternal temperature, and 29 trials enrolling participants without epidural labor analgesia.

**Table 1:** The characteristics of included studies

Study	Country	Maternal age (years) mean (SD)	gestational age (weeks) mean (SD)	RPCA	EA
Logtenberg 2017 [15]	Nertherlands	RPCA: 31.7 (3.9) EA: 31.8 (4.2)	RPCA:36.1 (34.3–37.6) EA:36.1 (33.9–37.7)	boluses:30 μg (20 to 40) lockout time:3 min no background infusion	Loading dose: 25 mg ropivacaine 0.2% CI: 0.1% ropivacaine plus sufentanil 0.5 μg/ml
Freeman 2015 [14]	Nertherlands	RPCA:31.5 (5.1) EA:31.7 (4.8)	RPCA: 37.8 (35.5–39.2) EA:37.1 (35.3–39.0)	boluses:30 μg (20 to 40) lockout time: 3 min no background infusion	epidural analgesia: ropivacaine or bupivacaine, or levobupivacaine plus sufentanil
Douma 2015 [16]	Nertherlands	RPCA:32 (4.8) EA:31 (5.6) Con:33 (4.5)	RPCA:39 EA:40 Con:40	bolus:40 μg lockout time: 2 min no background infusion	loading dose:25 mg (12.5 ml ropivacaine 0.2%) CI: ropivacaine 0.1% plus sufentanil 0.5 μg/ml
Stocki 2014 [17]	Israel	RPCA:31(5) EA:30 (6)	not reported	bolus:20-60 μg lockout time:2 min no background infusion	loading dose: 0.1% bupivacaine with 50 μg fentanyl 15 ml CI: 0.1% bupivacaine with 2 μg/ml fentanyl, PCA 10 ml lockout interval: 20 min
Ismail 2012 [21]	china	RPCA:28.35(5.54) EA:28.6 (5.49) CSEA:28.8 (5.50)	RPCA:39.2 (1.1) EA:39.0 (1.3) CSEA:39.1 (1.2)	bolus: 25 μg CI:0.1–0.9 μg/kg lockout time: 1 min	loading dose: 8 ml 0.125% levobupivacaine with 2 μg/mL fentanyl CI: 0.125% levobupivacaine with 2 μg/ml fentanyl, 8 ml/h CSEA: 2 mg levobupivacaine and 15 μg fentanyl (total 2 ml)
Evron 2008 [20]	Israel	RPCA: 29 (7) EA: 28 (5) RPCA+EA: 27 (5) RPCA+acetaminophen: 27 (4)	not reported	bolus: 20 μg lockout time: 3 min no background infusion	loading dose: 5–10 ml of 0.2% ropivacaine CI: 10 mg/h 0.2% ropivacaine PCEA: 10 mg 0.2% ropivacaine lockout: 20 min

*RPCA* remifentanil patient-controlled analgesia, *EA* epidural analgesia, *CSEA* combined spinal with epidural analgesia, *Con* control, *CI* continuous infusion.

All included RCTs compared RPCA with EA conducted by continuous infusion. As shown in Table 2, 2344 participants were actually received pain relief during labor. Three trials compared intravenous RPCA with EA [14, 15, 17]. One trial had two control groups including an EA group and a no analgesia group [16]. One trial assigned EA and combined spinal-epidural analgesia as the control groups [21]. The trial published by Evron et al. compared RPCA with three control groups including EA, RPCA plus EA, and RPCA plus acetaminophen [20].

**Table 2:** The participants and outcome of included study.

Study	randomised participants	drop-out	cross-over	actually received pain relief	intenstion-to-treat	Intrapartum fever definition	temperature mode	measure duration
Logtenberg 2017 [15]	418	RPCA:98 EA:105	RPCA:11 EA:14	RPCA:94 EA:76	yes	> 38 °C	not reported	during analgesia
Freeman 2015 [14]	1414	RPCA:22 EA:31	RPCA:53 EA:33	RPCA:447 EA:347	yes	> 38 °C	not reported	during labor
Douma 2015 [16]	116		RPCA:8 EA:1		no	≥38 °C	tympanic membrance	within 4 h
Stocki 2014 [17]	40	RPCA:1 EA:0	RPCA:3 EA:1	RPCA:19 EA:20	no	not reported	not reported	within 1 h
Ismail 2012 [21]	1140	RPCA:0 EA:0	not report	RPCA:380 EA:380 CSEA:380	no	not reported	oral temperature	within 1 h
Evron 2008 [20]	213	12	not report	201	no	≥38 °C	oral temperature	within 6 h

*RPCA* remifentanil patient-controlled analgesia, *EA* epidural analgesia, *CSEA* combined spinal with epidural analgesia, *Con* control, *CI* continuous infusion.

The characteristics of the included studies are reported in Table 1. There was no difference between RPCA and EA in maternal age and pregnant weeks. Six inclusion trials reported the randomized allocation, concealment regimen and sample size calculation. The participants and outcomes of the included studies are described in Table 2. Five trials were funded by nonprofit organizations [14, 16, 17, 20, 21], while no pharmaceutical company funded any trial.

**Fig. 2:**
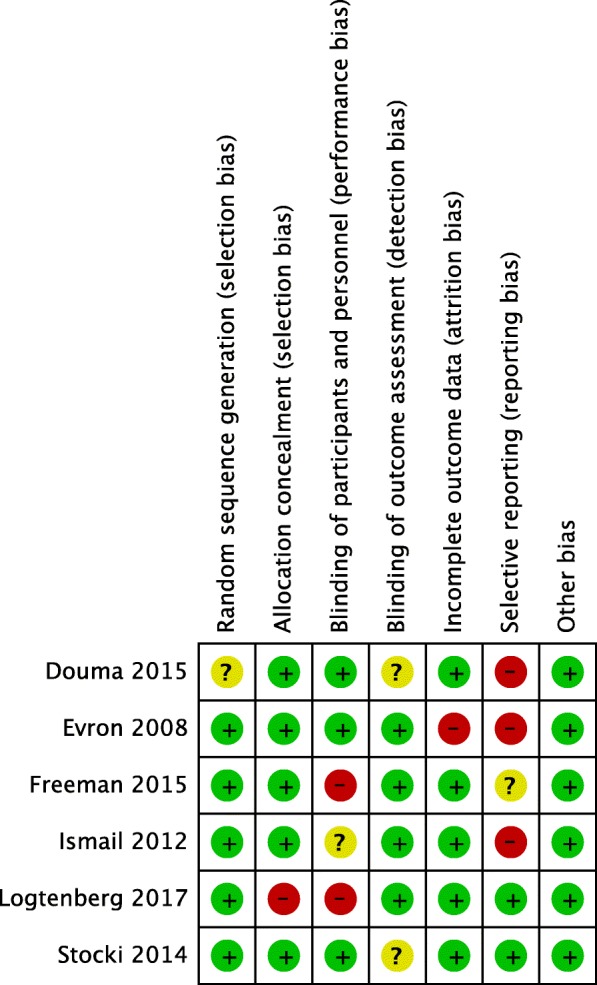
Risk of bias summary

The overall quality was good for all included studies. The risk of bias summary for each study is reported in Fig. 2, and the risk of bias graph for the individual trials were summarized in Fig. S1. The publications were found to have no bias according to the analysis of funnel plots in Fig. S2.

### Primary endpoint: the number of intrapartum maternal fevers

#### RPCA versus EA

**Fig. 3:**
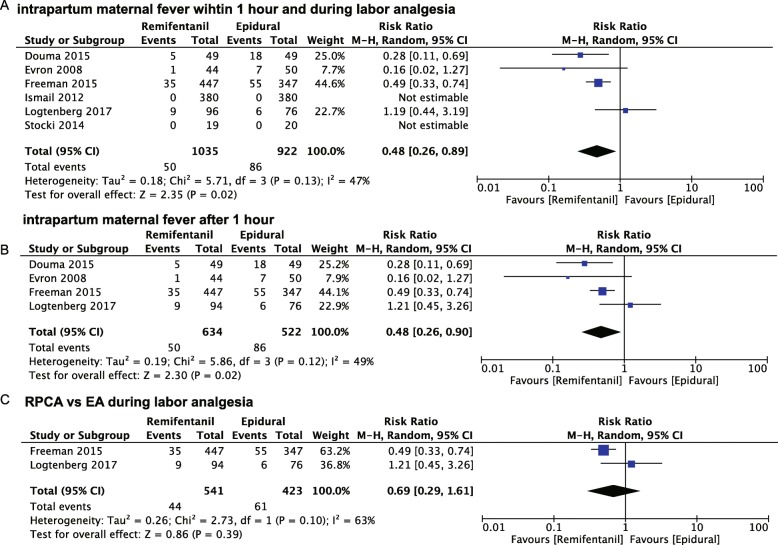
The effects of intravenous remifentanil patient-controlled versus epidural analgesia on intrapartum maternal fever within 1 h of (**a**), after 1 h of (**b**), and during labor analgesia (**c**)

Overall, six trials compared intravenous RPCA (*n* = 1035) with EA (*n* = 922) for labor pain relief, as shown in Fig. 3a [14–17, 20, 21]. The incidence of maternal fever with intravenous RPCA (50/1035) was lower than that with EA (86/922) during labor. The risk of intrapartum maternal fever with RPCA declined to 52% of that with EA (relative ratio 0.48, 95% confidence interval: 0.26–0.89, *P* = 0.02, I^2^ = 47%) within 1 or more hours. It drew our attention that the fever number of participants with fever in both the RPCA and EA groups remained at zero within 1 h of receiving pain relief. Thus, we excluded one trial by Ismail et al. [21] and another by Stocki et al. [17] to conduct the subgroup analysis. Compared with the risk of intrapartum maternal fever with EA, that with RPCA was 48% (relative ratio 0.48, 95% confidence interval: 0.26–0.90, *P* = 0.02, I^2^ = 49%) (Fig. 3b). Excluding two trials [16, 20] that not only investigated EA but also other analgesia modes other than RPCA increased heterogeneity from 49 to 63% (Fig. 3c). Those two trials [14, 15] remained to be assessed as having a high risk of bias for blinding (Fig. 2). Two studies (*n* = 964) demonstrated that there was no significant difference between RPCA and EA during the entire labor stage (relative ratio 0.69, 95% confidence interval: 0.29–1.61, *P* = 0.39. I^2^ = 63% )[14, 15](Fig. 3c).

### Secondary endpoints

#### Satisfaction with pain relief during labor

**Fig. 4:**
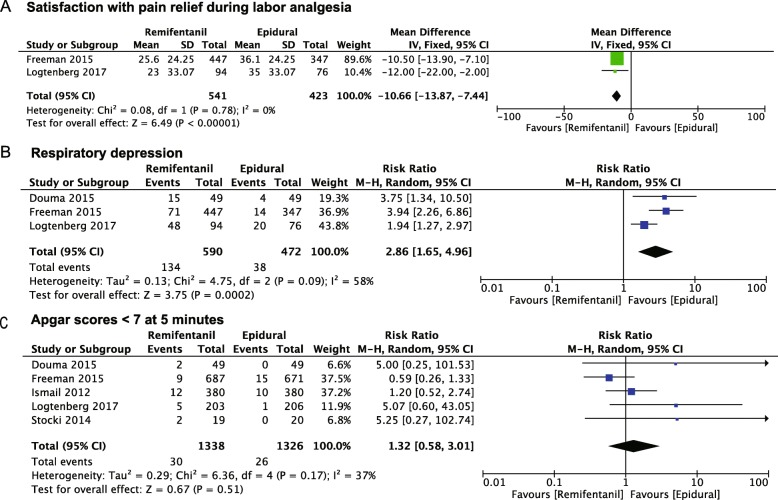
The effects of intravenous remifentanil patient-controlled versus epidural analgesia on the satisfaction with pain relief during labor analgesia (**a**), respiratory depression (**b**), and Apgar scores < 7 at 5 min (**c**)

Satisfaction with pain relief during labor is usually measured using a visual analog scale ranging from 0 to 100 mm. The AUC was a summary measure that integrated serial visual analog scale assessments, which represented satisfaction with pain relief at least two different time points. A higher AUC means a higher satisfaction with pain relief. Two trials (*n* = 954) compared RPCA with EA with respect to satisfaction with pain relief during labor analgesia using the AUC [14, 15]. The intention-to-treat was used to analyze the AUC in these two studies. As shown in Fig. 4a, RPCA was inferior to EA (− 10.6 [13.87, − 7.44], *P* < 0.00001, *I*^*2*^ = 0%).

#### Respiratory depression

Respiratory depression is thought to be the main complication related to remifentanil during labor analgesia. Three studies reported respiratory depression with pain relief during labor [14–16]. Compared with respiratory depression developed with EA, the incidence of respiratory depression significantly increased with RPCA (risk ratio 2.86 [1.65, 4.96], *P* = 0.0002, *I*^*2*^ = 58%) (Fig. 4b).

#### Apgar score at 5 min

The Apgar score at 5 min was reported in five of the included trials [14–17, 21]. No significant difference was demonstrated between RPCA and EA in the Apgar score < 7 at 5 min (risk ratio 1.32 [0.58, 3.01], *P* = 0.51, I^2^ = 37%) (Fig. 4c).

## Discussion

This systematic review and meta-analysis showed that the present data are insufficient to draw the conclusion that the incidence of intrapartum maternal fever is lower with intravenous RPCA than that with EA. Participants receiving intravenous RPCA experienced satisfaction with pain relief inferior to that of those receiving EA during labor. Meanwhile, respiratory depression occurred in patients receiving intravenous RPCA more frequently than it did in EA patients. There is an equivalent incidence of Apgar scores less than seven at 5 min between patients receiving intravenous RPCA and those receiving EA.

Regardless of when maternal temperature was measured, the risk ratio of intrapartum maternal fever after receiving intravenous RPCA was far lower than that after receiving EA. Within 1 h after pain relief, no maternal fever was observed in two studies [17, 21]. A previous study had investigated whether patients receiving EA developed pyrexia after only 6 h of labor [22]. Consistent with these finding by Fusi et al. [22], patients receiving EA with or without fentanyl are at an increased risk of hyperthermia approximately 5 h after analgesia compared with those receiving only parental opioid analgesia [23]. In addition, no such trend was observed in participants receiving such parental opioids as pethidine [22] or nalbuphine [23]. Obviously, an insufficient duration of measuring maternal temperature might not have allowed for a pyrexia event to be observed in the two trials [17, 21].

In response to excluding trials with other comparison groups, there was no significant difference in intrapartum maternal fever between the intravenous RPCA and EA groups. A good explanation is that the sample size was calculated according to the satisfaction with pain relief during labor [14, 15]. Furthermore, two studies reported that less than 50% of randomized participants actually received pain relief with RPCA or EA, resulting in a high selection bias. The primary endpoint was defined as the intrapartum maternal fever within 4 h in only one included study [16]. Regarding the limitations of such included trials, the current data fail to confirm that RPCA is superior to EA in decreasing the risk of intrapartum maternal fever. Future efforts towards this issue should mainly be focused on providing compelling data based from high-quality trials.

It is well established that the visual analog scale is usually used to assess pain relief. Actually, the intensity of pain is associated with the strength of uterine contractions during labor. The visual analog scale at a single timepoint is difficult to use to evaluate pain relief for the whole labor period, given that patients are not examined during a uterine contraction. The AUC based on a series of visual analog scales was used to compare the satisfaction with pain relief in this study. In contrast to women receiving EA, women receiving intravenous RPCA experienced the worst pain relief during labor, which was in agreement with a Cochrane review [24]. Interestingly, a previous clinical trial has demonstrated that intravenous remifentanil combined with epidural analgesia provides higher satisfaction with labor pain relief than single epidural analgesia [25]. A new multicenter, open-label, randomized controlled trial confirmed that RPCA is superior to pethidine for alleviating labor pain without serious adverse events [13]. Due to genetic polymorphisms, there are differences in not only opioid consumption but also in individual sensitivity to pain [26, 27]. If the individual genetic polymorphism is identified before administering opioids, intravenous remifentanil analgesia should yield better satisfaction with labor pain relief.

Respiratory depression in mothers and neonates is recognized as the main potential complication of remifentanil analgesia for labor pain. Our analysis demonstrated that patients receiving RPCA are at an increased risk of respiratory depression in comparison with those receiving EA. Respiratory depression was identified using the monitored desaturation. In fact, it is difficult to continuously monitor desaturation during labor. Hence, intravenous RPCA contributes to a much greater workload than EA. Fortunately, there were no significant effects on Apgar scores less than seven at 5 minutes by RPCA or EA. Intravenous RPCA is safe for women during delivery if frequent and careful desaturation monitoring is mandatory.

There are some limitations in this review. First, the direct comparison between RPCA and EA in maternal pyrexia during labor was only performed in one included trial. Second, the sample size of all included trials, except one by Douma et al. [16], was calculated according to such primary outcome as the intrapartum maternal fever during labor analgesia. There is no doubt that a high risk of analysis bias was seen among those included trials. Third, due to the different approaches of RPCA and EA, it is still difficult to achieve real blinding of both patients in labor and analgesia managers. A medium to high risk of performance and detection bias was seen in most of the included studies. The endpoint must be affected by the patient preference to avoid an invasive epidural catheter near to the spinal cord. It seems to be feasible to administer intravenous and epidural pumps in the same patient. However, it involves in an extra risk for the invasive epidural procedure in patients randomized to receive intravenous analgesia.

## Conclusion

This systematic review and meta-analysis suggested that there is no solid evidence to illustrate that the risk of intrapartum maternal fever decreases with RPCA in comparison with EA. Intravenous remifentanil analgesia is effective in attenuating labor pain. The increasing incidence of respiratory depression is an obstacle for utilizing intravenous remifentanil analgesia for pain relief during labor.

## Supplementary information

**Additional file 1.** The risk of bias graph for the individual trials.

**Additional file 2.** The funnel plot.

**Additional file 3.** Search strategy.

## Data Availability

All data generated or analyzed during this study are included in this article (and its supplementary files).

## Abbreviations

AUC: Area under the curve
EA: Epidural analgesia
ERMF: Epidural-related maternal fever
PRISMA: Preferred Reporting Items for Systematic Reviews and Meta-Analyses
PROSPERO: International Prospective Register of Systematic Reviews
RCTs: Randomized controlled trials
RPCA: Remifentanil patient-controlled analgesia
