# Supplementary material for: Remifentanil patient-controlled versus epidural analgesia on intrapartum maternal fever: a systematic review and meta-analysis
Source: BMC Pregnancy Childbirth. 2020 Mar 12;20:151. doi: 10.1186/s12884-020-2800-y (PMC7069013; doi:10.1186/s12884-020-2800-y)

**Search strategy:**

1. CENTRAL:

#1 (pain):ti,ab,kw OR (analgesia):ti,ab,kw OR (analgesic):ti,ab,kw (Word variations have been searched)

#2 (intrapartum):ti,ab,kw OR (maternal):ti,ab,kw OR (maternity):ti,ab,kw OR (labor):ti,ab,kw OR (labour):ti,ab,kw (Word variations have been searched)

#3 (temperature):ti,ab,kw OR (fever):ti,ab,kw OR (heat):ti,ab,kw OR (hyperthermia):ti,ab,kw OR (pyrexia):ti,ab,kw (Word variations have been searched)

#4 #1 AND #2 AND #3

#5 (intrapartum):ti,ab,kw OR (maternal):ti,ab,kw OR (maternity):ti,ab,kw OR (labor):ti,ab,kw OR (labour):ti,ab,kw (Word variations have been searched)

#6 (delivery):ti,ab,kw OR (parturition):ti,ab,kw (Word variations have been searched)

#7 #5 OR #6

#8 #1 AND #3 AND #7

1. Pubmed

| Search (((((analgesia[Title/Abstract]) OR analgesic[Title/Abstract]) OR pain[Title/Abstract])) AND (((((((intrapartum[Title/Abstract]) OR maternal[Title/Abstract]) OR maternity[Title/Abstract]) OR labor[Title/Abstract]) OR labour[Title/Abstract]) OR delivery[Title/Abstract]) OR parturition[Title/Abstract])) AND (((((temperature[Title/Abstract]) OR fever[Title/Abstract]) OR heat[Title/Abstract]) OR hyperthermia[Title/Abstract]) OR pyrexia[Title/Abstract]) |
| --- |
| Search ((((temperature[Title/Abstract]) OR fever[Title/Abstract]) OR heat[Title/Abstract]) OR hyperthermia[Title/Abstract]) OR pyrexia[Title/Abstract] |
| Search ((((((intrapartum[Title/Abstract]) OR maternal[Title/Abstract]) OR maternity[Title/Abstract]) OR labor[Title/Abstract]) OR labour[Title/Abstract]) OR delivery[Title/Abstract]) OR parturition[Title/Abstract] |
| Search ((analgesia[Title/Abstract]) OR analgesic[Title/Abstract]) OR pain[Title/Abstract] |

1. EMBASE


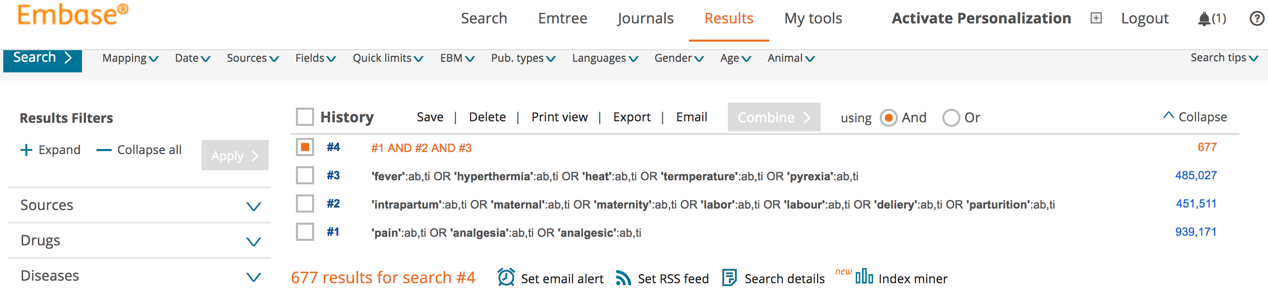

Supplement: Supplementary file 3 — Additional file 3. Search strategy. [file 12884_2020_2800_MOESM3_ESM.docx]
